# Genome instability goes mobile: damaged DNA transfer between human cells

**DOI:** 10.1038/s41392-026-03030-7

**Published:** 2026-10-09

**Authors:** Ashutosh Choudhury, Mathilde Courtes, Nitika Taneja

**Affiliations:** 1 Department of Molecular Genetics, Erasmus University Medical Center, Erasmus MC Cancer Institute, Rotterdam, The Netherlands; 2 Oncode Institute, Rotterdam, The Netherlands

**Keywords:** Genomic instability, Cell biology

In a recent study published in *Cell*, Maurais and colleagues^1^ identify a new dimension of genome instability heritability between human cells. The paper’s central message is striking: genomic instability generates cytoplasmic micronuclei and chromosome fragments, direct cell-cell contact enables their transfer, and transferred DNA can persist, function, and confer heritable phenotypic traits, including drug resistance.

Genome instability is recognized to impact the fate of neighboring cells, primarily through bystander effects. Although horizontal transfer of oncogenic DNA between cells has been described through uptake of apoptotic bodies or extracellular vesicles, direct contact-dependent transfer of damaged genomic DNA followed by its stable and functional maintenance in recipient human cells had not been established. Micronuclei arise from chromosome segregation errors, replication stress, and impaired repair. These fragile nuclear-like structures often undergo envelope rupture, exposing DNA to the cytoplasm and triggering cGAS–STING signaling, autophagic clearance or chromothripsis.^2^ This study identifies another possible fate of micronuclei as transferable cargo that can be maintained in adjacent cells. Can micronuclei therefore become vehicles propagating instability between cells? If micronuclear DNA can affect neighboring cells, then a genomically unstable cell may alter stability of nearby cells without classical clonal expansion.

A major strength of this paper is its careful experimental strategy to distinguish true transfer from imaging artefacts, chromatin bridges, or cell fusion. Authors first performed live-cell imaging of hTERT-immortalized human RPE1 cells treated to accumulate mitotic division errors and micronuclei. They observed DNA moving through connective structures resembling tunneling nanotubes (TNTs).

To confirm these observations, they used a color-coded chromatin system by co-culturing H2B-GFP- and H2B-mCherry-labeled cells. This elegantly showed donor-derived chromatin in recipient cells, appearing with a mismatched label. Plasma membranes were labeled using CAAX- HaloTag to delineate nanotube connections and confirmed DNA transfer across same and different epithelial cell types, including RPE-1 exchange with RPTEC cells. The work was further strengthened by using multiple sources of genome instability, including mitotic inhibitors, Mad2 depletion, nocodazole release, Cas9-induced breakage, and ionizing radiation. This suggests that DNA transfer is a broad consequence of cytoplasmic genome mis-localization.

**Fig. 1:**
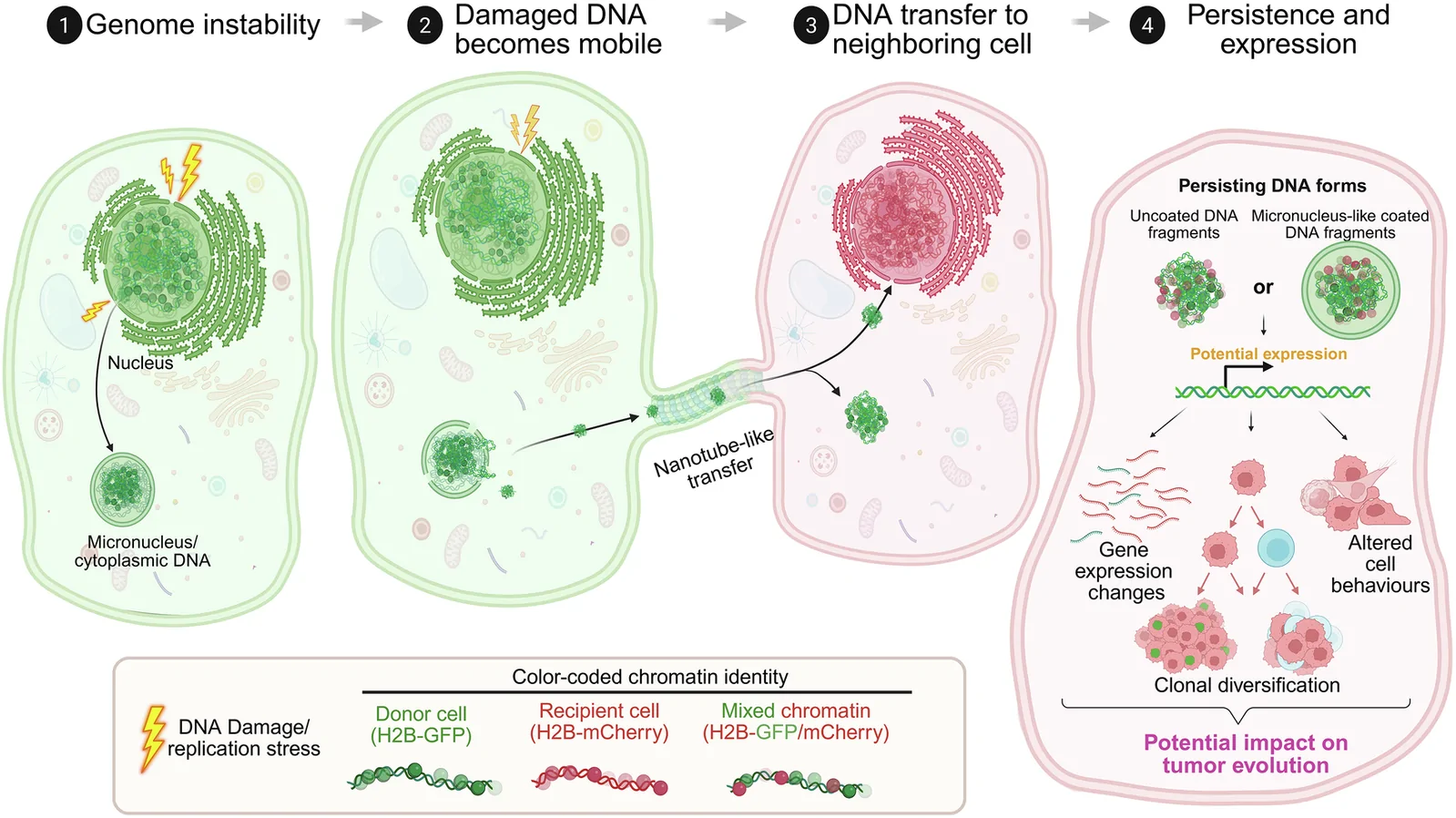
A new dimension of genome instability through horizontal propagation. DNA damage or replication stress can lead to genomic instability and the formation of free cytoplasmic DNA fragments, including DNA compartmentalized within micronuclei. Following micronuclear envelope rupture, damaged extranuclear DNA may become mobilized and transported from a donor cell (green) to an adjacent recipient cell (red) through distinct tunneling nanotube-like connections. Inside the recipient cell, the transferred DNA fragments, whether still encapsulated or uncoated, may persist as extrachromosomal DNA-like structures, intermingle with recipient-cell genetic material, and contribute to altered gene expression or downstream phenotypic changes. In the tumor environment, such instability-driven transfer of nuclear material could promote clonal diversification and influence cellular behaviors such as survival, proliferation, and migration. Image created with BioRender (https://app.biorender.com/)

The authors observed that only a subset of micronuclei was Lamin B1-positive, indicating that uncoated micronuclei can also transfer. In longer-term co-cultures, most transferred micronuclei acquired mixed donor and recipient H2B signals, suggesting incorporation of recipient histones during replication (Fig. 1). Does the recipient cell remodel or selectively maintain acquired DNA? Is maintenance impacted by micronuclei-coating status?^3^ Could micronuclei promote genotoxic and inflammatory signaling in recipient cells? Persistent cGAS–STING activation, together with ongoing replication stress, could trigger nuclear instability and adaptive genome reorganization, a process recently linked to replication-fork protection under stress.^4^ It may shape tumor evolution, therapy tolerance or de novo resistance in previously drug-naïve neighboring cells. Whether transferred DNA promotes such adaptive nuclear genome changes warrants further study.

Most compelling is the demonstration that transferred DNA can remain functional. To test this, the authors engineered male DLD-1 colorectal cancer donor cells carrying a neomycin-resistance cassette in the Y chromosome. Upon induced Y chromosome mis-segregation, co-cultured female HeLa recipient cells carrying a distinct antibiotic-resistance cassette acquired donor-derived Y chromosome fragments and G418 resistance, confirming de novo drug resistance from DNA movement.

This experiment converted an otherwise difficult-to-detect DNA-transfer event into a selectable phenotype. Coupled with FACS, karyotyping, SABER-FISH, and single-cell RNA sequencing, it shows that Y- derived fragments could persist as extrachromosomal elements and support gene expression. Importantly, these fragments appeared in cells whose chromosome number remained largely unchanged, arguing against simple cell fusion.

Thus, transferred DNA was not merely passive debris: it was maintained, transcriptionally active, and phenotypically consequential. Although micronucleus formation is likely stochastic, the persistence of transferred genomic fragments carrying functional genes, regulatory elements, or resistance-associated loci could create recipient cells with selectable adaptive traits, including drug resistance.

The authors appropriately position this process as horizontal gene transfer in mammalian cells. While horizontal gene transfer is well established in bacteria, and oncogenic or extracellular DNA transfer has been described in mammalian systems, the direct contact-dependent movement of genomic fragments between human cells and their stable inheritance as functional genetic elements had remained unclear. This study provides direct evidence that nuclear fragments can move between human cells and become inherited elements (Fig. 1).

In cancer, this could (i) contribute to clonal heterogeneity by introducing new copy-number changes or extrachromosomal DNA elements into recipient cells and (ii) provide a route for dissemination of oncogenic or resistance-associated DNA within a tumor ecosystem.^5^ Such events could arise intrinsically from chromosomal instability and replication stress or be enhanced following chemotherapy.

The extent to which intercellular DNA transfer influences tumor resistance remains to be determined. However, the study establishes a significant proof-of-principle: damaged genomic material can cross cellular borders and generate heritable traits, which may become consequential under strong selection.

Several key questions emerge: What determines whether cytoplasmic DNA is sensed by cGAS–STING, reincorporated into chromosomes, maintained as extrachromosomal DNA, or transferred to another cell? Is DNA cargo-selected, or is transfer largely stochastic? Which cytoskeletal motors and nanotube components drive DNA movement, and does the nature of the fragment affect transfer efficiency?

The observation that iPSCs show higher transfer is also intriguing, especially as it may relate to ROCK-inhibitor-driven enhanced nanotube formation. Could specific culture conditions, tumor states, or therapies similarly promote TNTs and intercellular genome transfer? Furthermore, could such transfers be rooted in normal human physiology and represent a cell-cell communication mechanism exploited by cancer cells? Whether intercellular DNA movement has physiological roles or instead reflects an incidental consequence of existing communication pathways remains an important question.

The study clearly reveals that damaged genomic material can cross cellular boundaries, persist in recipient cells, and confer new heritable traits. By combining live-cell imaging, differentially labeled chromatin, engineered chromosome mis-segregation, antibiotic selection, cytogenetics, and single-cell transcriptomics, the authors provide compelling evidence for intercellular genomic DNA transfer in human cells.

This work changes how we think about genome instability. Damaged DNA is not necessarily confined to the cell in which it arises. Under certain conditions, it can become mobile, functional, and phenotypically consequential in neighboring cells. This discovery opens a new field at the intersection of micronuclear biology, cell-cell communication, cancer evolution, and de novo drug resistance.
